# Application of Multivariate Statistical Methods to Optimize Water Quality Monitoring Network with Emphasis on the Pollution Caused by Fish Farms

**Published:** 2017-01

**Authors:** Mitra TAVAKOL, Reza ARJMANDI, Mansoureh SHAYEGHI, Seyed Masoud MONAVARI, Abdolreza KARBASSI

**Affiliations:** 1. Dept. of Environmental Sciences, Faculty of Environment and Energy, Science and Research Branch, Islamic Azad University, Tehran, Iran; 2. Dept. of Medical Entomology and Vector Control, School of Public Health, Tehran University of Medical Sciences, Tehran, Iran; 3. Dept. of Environmental Health Engineering, School of Public Health, Tehran University of Medical Sciences, Tehran, Iran

**Keywords:** Monitoring network, Water quality, Optimization, Environment, Statistics

## Abstract

**Background:**

One of the key issues in determining the quality of water in rivers is to create a water quality control network with a suitable performance. The measured qualitative variables at stations should be representative of all the changes in water quality in water systems. Since the increase in water quality monitoring stations increases annual monitoring costs, recognition of the stations with higher importance as well as main parameters can be effective in future decisions to improve the existing monitoring network.

**Methods:**

Sampling was carried out on 12 physical and chemical parameters measured at 15 stations during 2013–2014 in Haraz River, northern Iran.

**Results:**

The results of the measurements were analyzed using multivariate statistical analysis methods including cluster analysis (CA), principal component analysis (PCA), factor analysis (FA), and discriminant analysis (DA). According to the CA, PCA, and FA, the stations were divided into three groups of high pollution, medium pollution, and low pollution.

**Conclusion:**

The research findings confirm applicability of multivariate statistical techniques in the interpretation of large data sets, water quality assessment, and source apportionment of different pollution sources.

## Introduction

In recent decades, population growth, increasing need for countries to protein, good quality of fish protein compared to other proteins, remarkable marine resources, job creation especially in rural communities, and have put a spotlight on aquaculture industry more than ever (1–4). Discharge of effluent from fish farms that contains a variety of pollutants can lead to direct and indirect risks to human health and the environment (5–9).

A large number of people through the use of contaminated water are prone to various kinds of diseases (10, 11).

The risk of surface water contamination makes inevitable measurement and monitoring of these resources. Monitoring Process includes programs and activities that checks the quality of water resources and performance of pollution reduction system within various time periods. Since quality monitoring requires the measurement of various parameters in the river course and requires spending much time and cost, therefore, the design of an optimal network that, can adequately determine the quality of river systems with minimum cost, would be of great importance. Surface water influenced by chemical, physical, and biological contaminations that eventually affects the receiving environment and human health (12, 13).

Surface water systems include rivers, lakes, reservoirs, estuaries, and coastal waters. Apart from the anthropogenic sources, the quality of surface water can be influenced by geology of areas under investigation (14).

Therefore, assessment of river water quality is of great importance because it directly influences public health (via drinking water) and aquatic life (via raw water). Water Quality Indices (WQI) has been developed in the recent years. These indices are based on parameters such as pH, DO, BOD, NO3, PO4, TDS, TSS, EC, coliform, etc. Nowadays, river pollution due to aquaculture activities and the resulting risks have made the design of optimal water monitoring networks as one of the important topics of research around the world. Long-term monitoring of the seabed in West Bay affected by aquaculture facilities using univariate and multivariate analyses, achieved an adaptive monitoring protocol. This protocol provided the possibility to monitor pollutants from aquaculture activities in the region with the lowest cost and with high efficiency.(15)

A remote monitoring system designed for aquaculture cages in the open ocean for real-time measurement of water quality parameters (16).

A biological warning system (BWS) monitored identified or unknown pollutants of aquaculture environments (17).

After extensive monitoring 21 fish farms in Italy, the remains of organ chlorine pesticides examined in fish species. According to those results, levels of pesticides were much lower than the standard limits (18).

Dissolved organic matters caused by aquaculture systems in the rivers of northern Patagonia, Chile. Large amounts of dissolved organic matters disposed from these systems could leave significant adverse effects on the riverine ecosystem in north of Patagonia (19).

Multivariate statistical analysis due to its characteristics can be an efficient and useful technique for understanding and analysis of river pollution. It is also useful in proper reasoning and decision making in the management of water quality.

## Materials and Methods

### Study area

Haraz Watershed with an area of 5100 km^2^ is situated in the northern part of the Alborz Mountains between the longitudes 35° 45′-36° 42′ N and the latitudes 51° 27′-52° 42′ E (20). Several streams join the Haraz River. The most important of which are Namarestaq with a flow rate of 3 m^3^/s and Nour with a flow rate of 3.5 m^3^/s. Average discharge of Haraz River is 940 MCM/yr (20).

The river length in the longest tributary is 148 km and the width varies from 50 m to 500 m along the river course. The narrowest part of the river is near the sea where the water depth increases. The slope of the river bed is about 1% in the plain, about 7–8% in the middle plain and the city of Amol, and about 12%–13% in upstream of the river (21).

### Research procedure

**Fig. 1:**
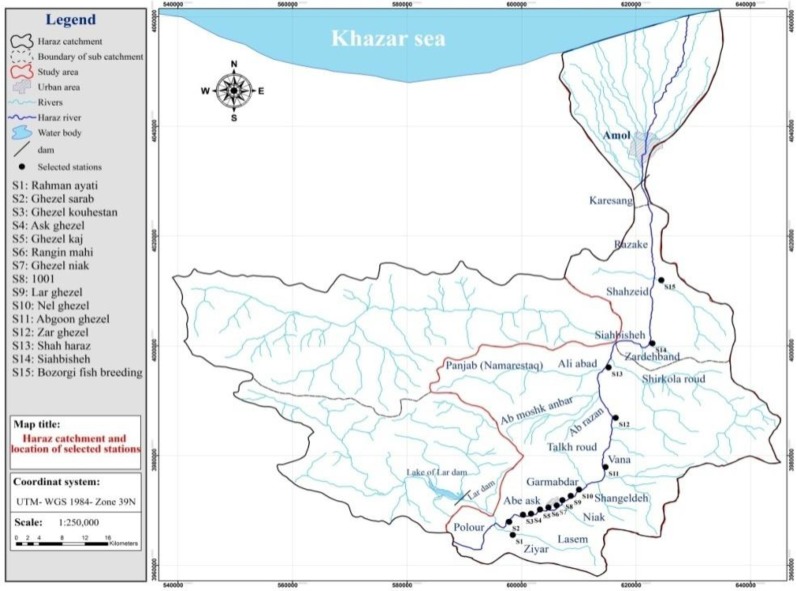
water quality mentoring stations in Haraz River

For sampling purposes, the maps of topography, geology, hydrology, pedology, land use, and access roads were prepared. Sampling stations were marked using Global Positioning System (GPS). The Samplings Stations were selected based on natural and manmade features such as river tributaries, geological structures, and pollution sources including farmlands, residential areas, industries, warm mineral springs, and fish ponds. Fig. 1 depicts distribution of the sampling station in the study area. Sampling was conducted in a one year time period from Sep 2013 to Sep 2014.

Table 1 provides a list of measurement methods and devices used in this research. A number of physiochemical parameters were measured on site using portable devices. The parameters such as temperature, dissolved oxygen, and pH were measured in-situ by portable device (model Sension156 Hach).

**Table 1:** measurement methods and devices used in the present research

**No.**	**Parameter**	**Abbreviation**	**Unit**	**Laboratory standard**	**Method or laboratory device**
	Dissolved oxygen	DO	mg/L	Standard method	Sension156 Hach
	pH	pH	pH unit	Standard method	Sension156 Hach
	Temperature	Temp.	c°	Standard method	Sension156 Hach
	Nitrate nitrogen	NO3-	mg/L	Nitrate with Test	Spectrophotometric
	Phosphate	PO43-	mg/L	Phosphate orth LR with Tube Test	Spectrophotometric
	Chemical oxygen demand	COD	mg/L	COD Total with Vario Tube	Dichromate reflex method
	Biochemical oxygen demand	BOD	mg/L	Instrumental method	Winkler azide method
	Total coliform	TColi.	MPN/10 0ml	A 9-tube system	Multiple tube method
	Fecal coliform	FColi.	MPN/10 0ml	A 9-tube system	Multiple tube method
	Discharge	Q	m3/s	Synoptic data	Synoptic data

Before statistical analyses, Kolmogorov-Smirnov test was used to check the normality and goodness of fit of the measured data.

Accordingly, a goodness of fit test for a normal distribution was conducted using parametric methods. Similarly, the Bartlett test was used to check the fitness of data for Principal component analysis (PCA) and along with factor analysis (FA).

To study the differences between the stations in terms of changes in various parameters, ANOVA (one-way or two-way) was used. Comparing the average of parameters with significant F between the stations was performed using Duncan test at probability of 5%. Subsequently, the stepwise regression coefficients were calculated to identify important parameters (independent variables) affecting the changes in any of the parameters (dependent variable). For the purpose of classification of variables, cluster analysis was performed based on Euclidean distance by Un-weighted Paired Group Method using Arithmetic Averages (UPGMA) and standardized variables. All statistical and mathematical analyses were performed using Excel 2007, SPSS 16, and MINI-TAB 15.

## Results

After calculating central tendency and dispersion of the parameters, their normality was checked using Kolmogorov–Smirnov test. The test results confirm the normal distribution of the data with a confidence coefficient of 95% (*P*-value ≥0.05) The results of the ANOVA and post hoc (Duncan) tests are provided in Table 2. According to the ANOVA test, there was no significant difference between the pH of various stations (*P*≥0.05).

**Table 2:** ANOVA and post hoc (Duncan) test results for comparison of the measurement parameters at different stations

**Parameter**		**Sum of Squares**	**df**	**Mean Square**	**F**	**Sig.**
DO	Between Groups	66.326	14	4.738	5.336	.000
	Within Groups	146.489	165	.888		
	Total	212.815	179			
Temp	Between Groups	996.436	14	71.174	2.823	.001
	Within Groups	4160.479	165	25.215		
	Total	5156.915	179			
pH	Between Groups	.095	14	.007	.031	1.000
	Within Groups	36.471	165	.221		
	Total	36.566	179			
BOD	Between Groups	46716.478	14	3336.891	2.921	.001
	Within Groups	188501.583	165	1142.434		
	Total	235218.061	179			
Phosphate	Between Groups	51.819	14	3.701	6.252	.000
	Within Groups	97.677	165	.592		
	Total	149.496	179			
Nitrate	Between Groups	548.158	14	39.154	28.147	.000
	Within Groups	229.527	165	1.391		
	Total	777.685	179			
COD	Between Groups	546697.633	14	39049.831	2.061	.016
	Within Groups	3125575.167	165	18942.880		
	Total	3672272.800	179			
Pb	Between Groups	4.644	14	.332	28.601	.000
	Within Groups	1.914	165	.012		
	Total	6.558	179			

Therefore, it was not required to use Duncan test for pairwise comparison of this parameter at different stations. Other parameters showed significant differences at different stations (*P*≤0.05). Moreover, Duncan test was used to compare pair wisely other parameters.

**Fig. 2:**
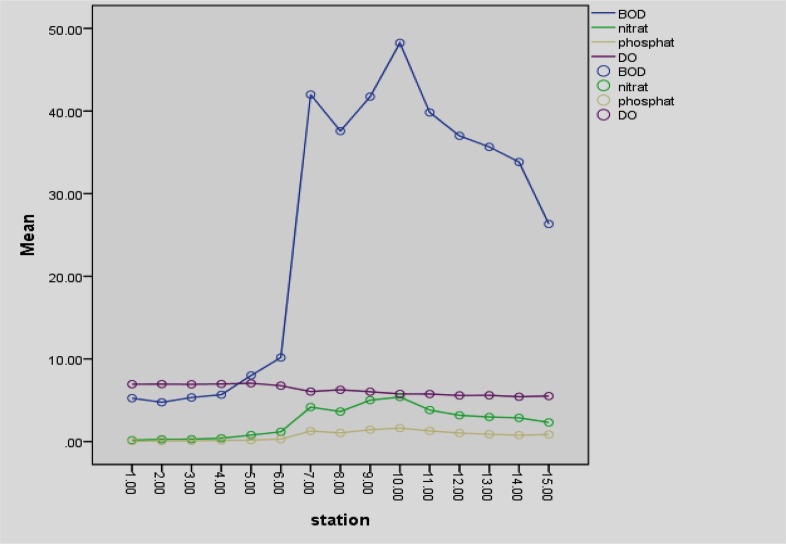
Changing trend of nitrate, phosphate, DO, and BOD over the various stations in Haraz River (mg/l)

The changing trend of nitrate, phosphate, DO, and BOD over the various stations is depicted in Fig. 2.

### Cluster analysis

Investigation of relationship between the 12 parameters and 15 stations requires correlation matrix of 12 × 12. After standardization of data, the cluster analysis was performed using UPGMA method based on least square Euclidean Distance. The parameters were clustered from 0 to 12 based on the re-scaled dissimilarity. For clustering of water quality data, the average value of parameters during the sampling periods was used. Dendrogram divided the stations in three clusters based on the farthest Euclidean distance.

**Fig. 3:**
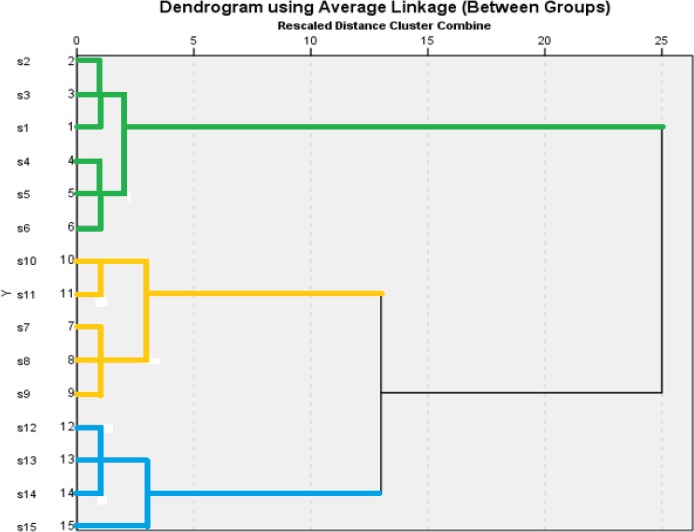
cluster dendrogram of the stations based on the measured parameters in Haraz River Basin

Fig. 3 depicts cluster analysis of the stations based on the measured parameters in Haraz River Basin. According to the dendrogram, the stations were divided into three clusters. The first cluster included stations 1 to 6 while the second cluster contained the stations 7 to 11. There are 4 stations (12 to 15) in the third cluster.

The stations in the third cluster were the most polluted stations. These places, in terms of pollution level, were distinct from other stations and were placed farthest from the rest. The pollution level of the stations in the second cluster was medium while the other stations grouped in the first cluster were low in contaminants. No significant difference was found between the parameters of the stations in each cluster while the difference was significant between the clusters, at probability levels of 1% and 5%. This revealed the fact that for rapid monitoring of water quality, it is just enough to select only one station at each cluster.

### Results of PCA and FA

According to the eigenvalues, high-value parameters in the first three components could justify 76% of the changes between the stations. Thus, the first and second main components, with eigenvalues of 14.44 and 3.98, account for 63.09% of changes between the stations.

The shares of each of the first and second components are 46.87% and 16.22%, respectively. Comparing eigenvalues of first and second components indicate that the first component has the highest share of changes in the model. Large differences between the stations are due to the parameters of discharge, coliform, DO, nitrate, phosphate, BOD, and temperature.

According to this component, Pb, COD, pH, and dyestuff had a lower contribution in variability and the difference between the stations.

In other words, the pollution of each station with higher PC_1_ is mainly affected by the activities of aquaculture, agricultural and industrial wastewater.

In the PC_2_, COD, pH, dyestuff, and Pb with high positive coefficients, and the parameters of discharge, coliform, turbidity, DO, nitrate, phosphate, temperature, and BOD with high negative coefficients showed a different response to other parameters. 46.87% of changes between the stations in PC1 is related to the higher coefficients in the first component while in PC2, 16.22% of the variations between the stations are related to the parameters with higher coefficients in the second component.

In PC1 and PC2, the coefficient of parameters discharge, coliform, DO, nitrate, phosphate, temperature, BOD was almost high. This indicates greater influence of these parameters on the difference between the stations.

Similar to the previously discussed results in cluster analysis, based on the two components of PC_1_ and PC_2_, the stations can be divided into three groups of high pollution (third group), medium pollution (second group), and low pollution (first group).

Accordingly, the results of PCA and cluster analysis are in good agreement. The results of the FA also confirm the findings of the PCA. Based on FA, 63.09% of the variances are justified by the first two factors. In other words, these two factors account for the most of the changes. According to the rotated component matrix (also called the rotated factor matrix), the parameters of discharge, coliform, DO, nitrate, phosphate, BOD, and temperature are under the influence of the first factor while the parameters of pH, COD, dyestuff, and Pb are affected by the second factor. Each variable is categorized in a factor that has a high significant correlation with it.

**Fig. 4:**
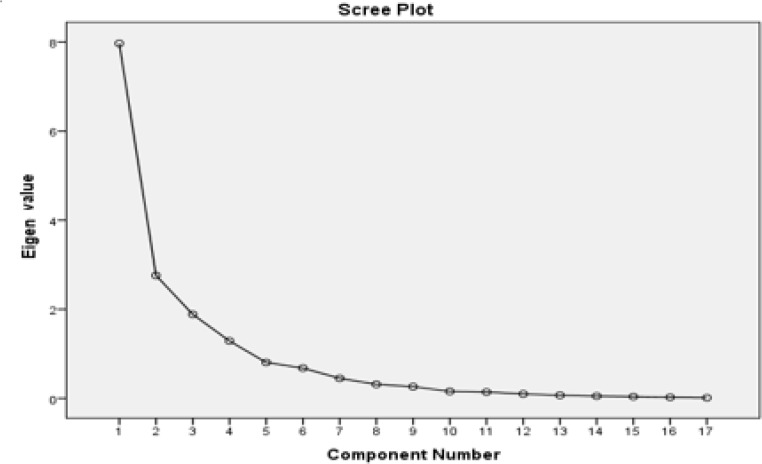
Relationship between the factors and eigenvalues

Fig. 4 illustrates the relationship between the factors and eigenvalues.

### Results of discriminant function

In this research, the discriminant analysis was done on the raw data of the groups derived from PCA, FA, and CA. Discriminant functions were calculated for all of the parameters in different groups.

The results show that 100% of the total variance is explained by the first two discriminant functions. The function alone accounts for 63.09% of the total variance. Thus, by using the first and second functions, those parameters that have the greatest impact on grouping of the stations could be assigned to different groups. Using these coefficients, it could be possible to derive a numerical value for each station according to relevant parameters. By the comparison of this value with those amounts provided for each group by the discriminant functions, stations can be categorized in groups that have the least distance from each other.

Wilks Lambda discriminant analysis results showed that the first and second rows are significant at the 99% probability level. After removing the effect associated with the first discriminant function, the results indicated a significant difference between the groups in terms of all parameters evaluated in the study area.

The value of Kappa coefficient in this study was +1, which confirms the accuracy of predictions. Furthermore, the KMO of 0.76 approved the possibility of using PCA and PFA.

## Discussion

Generally, the present study showed the application of multivariate statistical analysis to optimize water quality monitoring networks. The results revealed that how statistical analyses are able to identify the optimum number of stations and determine the key parameters. When data are from a large number of variables and sources of change (stations and periods), one of the applicable methods of careful data analysis is the use of multivariate techniques to reduce the data volume by taking into consideration the relationship between the variables. Another commonly-used technique is PCA.

The purpose of this analysis is to reduce the volume of data so that they contain almost all aspects of the original data. In other words, the purpose of the PCA is to describe a set of correlated data in the form of a set of uncorrelated variables that are a linear combination of the variables.

In a study with a combination of ANOVA and multivariate statistical methods redesigned a water quality-monitoring network. They concluded that these methods are very efficient in determining the measurement parameters (22).

In another study, multivariate statistical methods in order to optimize a water quality monitoring network in the Klang River Basin in Kuala Lumpur. Using the statistical analysis, they managed to identify important and effective parameters for water monitoring in the basin (23).

The application of multivariate statistical analysis examined in assessment of water quality of the Tigris River in Iraq. They also reported similar results regarding the efficacy of this analysis to optimize the monitoring network (24).

The water quality of a large dam assessed in Brazil based on multivariate statistical methods, evaluated these techniques very effective in reducing the number of sampling pints, and subsequently a substantial decrease in the cost of testing (25).

The applicability of multivariate statistical analysis confirmed in determining spatial and temporal frequencies of water quality measurements in White River Basin in USA (26).

## Conclusion

The present study showed the applicability of multivariate statistical methods in determining key water quality parameters and optimal number of measurement stations. This method can be very useful in studies of water quality, when data is from a large number of variables and sources of change (stations). These methods, taking into account the relationship between the variables, can identify the main stations and parameters that are responsible for the most of changes in the target system. This will have a significant role in saving time and cost of measures whereas one of the most important issues in determining the quality of water in rivers is to create an optimal water quality control network so that the measured variables could be representative of the entire changes in the water quality.

Determine the main parameters and stations with higher importance can be effective in future decisions to improve the existing monitoring networks, to remove or add stations and new parameters, and to update the sampling frequency.

Using this method will help researchers identify the most important factors affecting the quality of water systems and is considered a valuable tool for assured management of water.

## Ethical considerations

Ethical issues (Including plagiarism, informed consent, misconduct, data fabrication and/or falsification, double publication and/or submission, redundancy, etc.) have been completely observed by the authors.
